# *Bacillus subtilis* immobilization on biochars produced from different feedstocks and pyrolysis temperatures: performance, mechanisms, and salt tolerance

**DOI:** 10.1186/s40643-026-01070-z

**Published:** 2026-06-03

**Authors:** Zhixiang Jiang, Bin Liu, Rui Chen, Liankai Zhang, Guiren Chen

**Affiliations:** 1 Kunming General Survey of Natural Resources Center, China Geological Survey, Kunming, 650111 China; 2 School of Environment and Geography, Qingdao University, Qingdao, 266071 China; 3 Yunnan Technology Innovation Center of Natural Resources Carbon Sink Investigation and Carbon Asset Assessment, Kunming, 650111 China

**Keywords:** Adsorption kinetics, Adsorption isotherm, Biochar-based microbial fertilizer, Biochar properties, Pyrolysis temperature, Salt tolerance test

## Abstract

Using biochar as a carrier to enhance the adaptability and survival of inoculated microorganisms under harsh environmental conditions is considered as a promising strategy. However, the immobilization performance and underlying mechanisms of microorganisms on different biochars remain insufficiently understood. In this study, a series of biochars were prepared from various feedstocks and at different temperatures, and their capacities to immobilize *Bacillus subtilis* (*B.subtilis*) were evaluated. The results demonstrated that high-temperature (700 °C) biochars exhibited 14.40%–60.00% greater loading capacity for *B.subtilis*, compared to low-temperature (400 °C) biochars. Analyses of surface morphology, functional groups, hydrophobicity, and Zeta potential before and after immobilization indicated that *B.subtilis* loading significantly altered the surface properties of biochar, including increases in C and N contents, enhanced richness and diversity of functional groups, and a transformation from hydrophobicity to hydrophilicity. Adsorption kinetics and isotherm modeling revealed that the immobilization process was dominated by chemical adsorption, characterized by monolayer adsorption on a homogeneous surface. Interface interaction analysis further confirmed that the electrostatic interaction, hydrogen bonding, and hydrophobic forces between the functional groups of biochar and those on *B.subtilis* cells synergistically facilitated microbial immobilization. Based on these findings, a two-stage adsorption process of *B.subtilis* on biochar was proposed: initial surface adhesion and pore filling, followed by extracellular polymer and surface functional group complexation. Biochar properties, including specific surface area, pore volume, Zeta potential, hydrophobicity, C/N ratio, and surface functional groups, were further identified as key factors influencing immobilization. Finally, the salt tolerance of *B.subtilis* was significantly enhanced when immobilized on biochars, particularly corn straw biochars with large specific surface areas and developed pore structures. This improved salt tolerance suggests that the biochar-based microbial fertilizers could serve as a promising approach for the amelioration of salt-alkaline soil.

**Graphical abstract:**

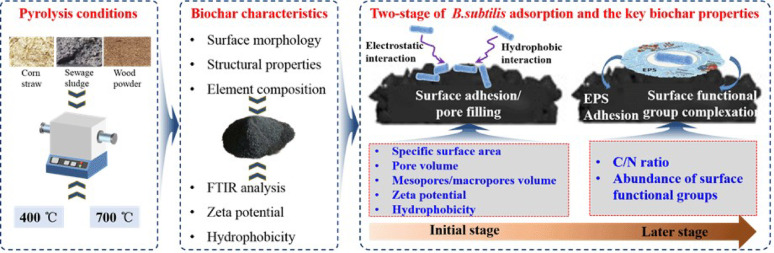

## Introduction

As the global population continues to grow, the escalating demand for food has led to increased pressure on arable land, resulting in significant degradation of soil quality, such as salinization (Bazihizina et al. 2024; Kopittke et al. 2019). This poses a serious threat to global food security. Salinization soils and other naturally saline-alkaline soils are important cultivated land resources, but their high salinity and alkalinity limit soil fertility and crop productivity (Liu et al. 2025). Therefore, improving the soil conditions of saline-alkaline lands and enhancing crop yields have become critical issues in contemporary agricultural development.

In recent years, microbial inoculants have demonstrated significant potential in mitigating the effects of salt stress on plant growth and have garnered widespread attention as a green soil remediation technology (Khan et al. 2021). These inoculants have been evidenced to effectively improve the properties of saline-alkali soils through various mechanism, thereby promoting plant growth (Jia et al. 2023). For instance, certain microbial inoculants can lower soil salinity and alkalinity by secreting organic acids, creating a more favorable alkaline environment for plant growth (Liu et al. 2025). Other inoculants decompose organic matter and minerals in the soils, enhancing nutrients availability and improving plant stress tolerance (Yuan et al. 2022). However, exogenously inoculated microbes are highly susceptible to the initial environmental conditions of the inoculated soils, such as high pH and salinity, low nutrient content, and competition from native microorganisms. These factors can negatively impact the survival and colonization rates of the inoculants, ultimately reducing their stability and efficacy (Du et al. 2022). To address this issue, some inoculated microbial enhancement techniques have been developed (Zhang et al. 2023), among which loading microbial inoculants onto suitable carriers has emerged as a promising approach. Carriers can shield inoculated microbes from adverse external environmental factors, thereby enhancing their colonization and effectiveness. Biochar, a cost-effective and readily available carbon-rich material, is considered as an ideal microbial carrier due to its porous structure, large surface area, abundant functional groups and specific charge capacity, as well as high chemical and mechanical stability (Gong et al. 2022). Additionally, biochar itself can promote plant growth by improving soil physical and chemical properties (Dai et al. 2020). Consequently, a considerable amount of studies has focused on developing “biochar-microbe” composite materials and testing their application performance in various fields (Bolan et al. 2023). However, research on the preparation and application of biochar-based microbial fertilizer (BMF) with high salt tolerance remains limited.

Currently, the preparation of BMFs mainly includes adsorption, encapsulation, crosslinking, and covalent binding (Du et al. 2022). Many studies have suggested that microbial attachment to biochar occurs through various processes/mechanisms, including surface adsorption, pore filling, electrostatic interactions, covalent bonding, and ionic grid formation (Bolan et al. 2023). These findings indicate that the loading capacity and mechanism of microbes on different biochars may vary substantially, as biochars prepared from diverse feedstocks and pyrolysis temperatures demonstrate distinct physicochemical characteristics (Xiong et al. 2017). However, a systematic understanding of the relative contributions of these mechanisms to overall microbial adsorption remains lacking. The dominant adsorption mechanisms are likely controlled by the intrinsic properties of biochar, such as surface area, porous structure, surface functional groups, pH, C/N ratio, etc., which are closely associated with feedstock type and pyrolysis temperature (Xiong et al. 2017). Previous studies have consistently shown that increasing pyrolysis temperature enhances biochar pH, electrical conductivity (EC), C/N ratio, ash content, Zeta potential, and specific surface area, while reducing the O/C and H/C ratios and the abundance of oxygen-containing groups (Ippolito et al. 2020; Ortiz et al. 2020; Tomczyk et al. 2020; Zhu et al. 2026). Regarding feedstock, wood-derived biochars typically exhibit higher specific surface area, pore volume, C content, and C/N ratio, whereas biochars derived from crop straw or other biowaste appear to have greater cation exchange capacity, pH, ash/mineral content, and a higher abundance of oxygen (O)-containing functional groups (Ippolito et al. 2020; Zhu et al. 2026). Despite these insights, a clear knowledge gap remains regarding how feedstock type and pyrolysis temperature, through their effects on biochar properties, influence microbial loading performance and the underlying mechanisms. Therefore, investigating the loading performances of inoculated microbes on a series of biochars produced from different feedstocks and pyrolysis temperatures and elucidating the underlying regulatory pathways of “preparation conditions → biochar properties → dominant adsorption mechanism” is urgently necessary. To address this knowledge gap, this study aims to: (1) quantitatively investigate the microbial loading efficiency of a series of biochars produced from different kinds of raw feedstock and pyrolysis temperatures; (2) explore the loading mechanism of microbes on biochars and identified the key biochar properties; and (3) determine the salt tolerance of different kinds of BMFs. Finally, the best BMFs that can be potentially applied to the improvement of saline-alkali soils is recommended. The findings presented in this study will provide strong scientific theoretical support for optimizing the preparation of BMF.

## Materials and methods

### Preparation and characteristics of immobilized *Bacillus subtilis (B.subtilis)* on biochar

*B.subtilis* strain was obtained from the laboratory of Qingdao University. The bacteria were cultured in LB medium with shaking at 170 rpm and incubated at 30 °C for 24 h. After incubation, cells in the logarithmic growth phase were harvested by centrifugation at 4000 rpm for 5 min, and washed three times with 0.85% physiological saline. The washed bacteria were then resuspended in 0.85% physiological saline. The microbial concentration in the bacterial suspensions was determined by measuring the optical density (OD) at 600 nm (OD_600)_ using a UV-2600 spectrophotometer (Shimadzu, Japan). During the logarithmic growth phase, a strong linear relationship between OD_600_ and colony-forming units (CFU mL^‒1^) (R^2^ > 0.98). The CFU values of these suspensions were further quantified using the standard dilution plate count method. An OD_600_ nm of 1.0 (~ 2 × 10^‒9^ CFU mL^‒1^) was used as inoculum for all experiments unless otherwise specified.

To investigate the effects of feedstock type on *B.subtilis* loading, three commonly used materials with distinct organic and inorganic compositions were selected: corn straw, wood powder, and sewage sludge. Corn straw is rich in cellulose and hemicellulose, wood powder contains a high lignin content, and sewage sludge exhibits low carbon content but high nutrient and mineral levels. These compositional differences are expected to significantly influence the physicochemical properties of the resulting biochars (Zhu et al. 2026). The materials were crushed by a pulverizer firstly. A vacuum tube furnace was employed to produce the biochars. In biochar research, pyrolysis temperature typically ranges from 300 to 900 °C, with 500 °C commonly regarded as the threshold between low- and high-temperature biochars, which differ markedly in in physicochemical properties. In this study, biochars produced at 400 °C and 700 °C were selected to represent low- and high-temperature biochars, respectively, to examine the effects of pyrolysis temperature-driven property variations on *B.subtilis* loading. Pyrolysis was performed at a heating rate of 10 °C min^–1^, with a 2-hour residence time at the peak temperature. The entire process, including post-pyrolysis cooling, was conducted under a N_2_ atmosphere. Finally, six biochars from corn stalks, wood powder, and sewage sludge at 400 °C and 700 °C were obtained, naming as CSB400, CSB700, WPB400, WPB700, SSB400, and SSB700, correspondingly.

In the present study, two BMF preparation methods, including adsorption and adsorption-encapsulation, were employed. For the adsorption method, each biochar sample was mixed with bacterial suspension at ratio of 1:20 (w/v) in a 250 mL conical flask and incubated at 30 °C with shaking at 170 rpm for 6 h. The resulting biochar-bacterium mixtures were then filtered using a 200-mesh cell sieve. For the preparation of adsorption-encapsulation-type BMFs, the same biochar-bacterium suspensions were further mixed in equal volume with a 3% (w/v) sodium alginate solution and shaken for 30 min. The resulting mixtures were dropped uniformly into a 2% (w/v) CaCl_2_ solution using a sterile syringe and allowed to crosslink for 12 h. Meanwhile, an encapsulated bacterial suspension without biochar was prepared as a control. All BMF samples recovered from the solution were freeze-dried and then characterized as soon as possible time. All operations, including filtration, were performed under sterile conditions to prevent contamination and ensure the integrity of the microbial communities. In total, six adsorption-type BMFs (ACB400, ACB700, AWB400, AWB700, ASB400, ASB700) and six adsorption-encapsulation-type BMFs (ECB400, ECB700, EWB400, EWB700, ESB400, ESB700) were obtained.

### Material characterization and analysis

The number of microorganisms loaded on each BMF was determined using the lipid-phosphorus method (Zhang and Wang 2021). The element constitute, including carbon (C), hydrogen (H), nitrogen (N), and sulfur (S), of original biochars and BMFs was measured using an elemental analyzer (Vario EL cube). Oxygen (O) content was calculated by difference method. The surface area and pore structure of the biochars were characterized by a surface area analyzer (ASAP 2460) at 77 K based on the Bruner-Emmet-Teller (BET) nitrogen adsorption method. The morphology and surface structure of the original biochars and BMFs were examined by scanning electron microscopy (SEM). Fourier transform infrared spectroscopy (FTIR; NICOLET 6700) was employed to identify functional groups in the original biochars and BMFs. The hydrophilicity and surface charge (Zeta potential) of the original biochars and BMFs were measured using a contact angle analyzer (Drop Shape Analyzer-DSA100) and a Zeta potentiometer (Nanoplous-3), respectively. The physicochemical properties of the initial biochars are summarized in Table 1.

**Table 1:** Basic properties of the different biochars

Parameter	SSB400	SSB700	WPB400	WPB700	CSB400	CSB700
pH	9.26 ± 0.03d	10.70 ± 0.12a	8.47 ± 0.14e	10.30 ± 0.06b	9.00 ± 0.03f	9.56 ± 0.04c
EC	137.20 ± 15.89d	356.33 ± 22.19c	331.00 ± 135.53c	848.33 ± 100.11a	539.00 ± 23.39b	635.67 ± 44.46b
N (%)	3.20 ± 0.01a	1.52 ± 0.01d	2.81 ± 0.01b	2.39 ± 0.01c	1.22 ± 0.01f	0.92 ± 0.01f
C (%)	26.11 ± 0.15 d	28.71 ± 0.20cd	64.51 ± 0.18a	75.03 ± 0.21a	36.53 ± 0.09c	42.77 ± 0.11b
H (%)	1.54 ± 0.01c	0.80 ± 0.01d	3.15 ± 0.07a	1.72 ± 0.04c	2.22 ± 0.05b	1.14 ± 0.03c
S (%)	2.38 ± 0.02a	2.43 ± 0.02a	0.43 ± 0.02b	0.50 ± 0.02b	0.46 ± 0.02b	0.44 ± 0.01b
C/H ratio	16.94 ± 0.20d	36.11 ± 0.25c	20.51 ± 0.07d	39.59 ± 0.13a	16.47 ± 0.07d	37.61 ± 0.18b
C/N ratio	8.16 ± 0.55f	18.89 ± 0.54d	22.96 ± 0.13c	31.39 ± 0.17b	29.94 ± 0.08b	46.49 ± 0.22a
BET Surface Area (m^2^ g^−1^ )	1.2824	2.5902	4.1276	14.4052	5.1833	49.5884
Mesoporous and macropore volumes(cm^3^ g^−1^ )	0.000268	0.000358	0.001786	0.006925	0.001785	0.007107
Average pore size (nm )	4050.52	4214.61	3869.75	1823.07	3795.68	1569.84

Data are represented as the means ± SD (standard deviation). The different lower case letters within the same line indicate significant difference between the treatments, which as analyzed by the Tukey’s T test (*p* < 0.05). Abbreviations: EC, electrical conductivity; SSB400 and SSB700, sewage sludge biochars produced at 400 °C and 700 °C, respectively; WPB400 and WPB700, wood power biochars produced at 400 °C and 700 °C, respectively; CSB400 and CSB700, corn stalk biochars produced at 400 °C and 700 °C, respectively

### Batch adsorption experiment

In order to explore the adsorption performance and mechanism of *B.subtilis* on biochar affected by biochar type, adsorption kinetics and isotherm experiments were designed. Regarding adsorption kinetics experiment, each kind of biochar (3 g) was added to 60 mL of overnight cultures (OD600 = 1.0 ± 0.05) and incubated in a 150 mL conical flask at 30 °C and shaken at 170 rpm for 0, 20, 40, 60, 120, and 180 min. At each time interval, take the mixture out of the conical flask and filter it through a 15-micron nylon filter paper. Then, the OD value of the sample was measured. For adsorption isotherm experiment, a series of *B.subtilis* suspensions with varying *B.subtilis* concentrations were prepared firstly as follows: overnight cultures were centrifuged and the cell pellets were resuspended in fresh LB medium. The suspensions were then adjusted to an OD600 value of 1.0 ± 0.05, corresponding to 100% concentration. Based on this stock solution, serial dilutions were prepared to achieve OD600 values of 0.8 ± 0.05, 0.6 ± 0.05, 0.4 ± 0.05, and 0.2 ± 0.05, corresponding to 80%, 60%, 40%, and 20% concentrations, respectively. A free LB solution was used as a control. Each kind of biochar (3 g) was mixed with the prepared *B.subtilis* suspensions, and then the solutions were shaken for 180 min to achieve adsorption equilibrium. For both adsorption kinetics and isotherm experiments, the pH of each suspension was adjusted to 7.00 ± 0.05 immediately after mixing with biochar by dropwise addition of 0.1 M HCl or 0.1 M NaOH with gentle stirring. Using the difference between the initial (C_o,_ CFU mL^‒1^) and final (C_f,_ CFU mL^‒1^) concentrations of *B.subtilis* in the supernatant to calculate the immobilization amount of *B.subtilis* on biochar (*qe*, CFU g^–1^) at a given point of time [Eq. (1)]:

1 $$\:{q}_{e}=\frac{V({C}_{0}-{C}_{f})}{M}$$

where V is the volume of cell suspension (mL) and *M* is the dosage of biochar added (g). Four models, including Pseudo-First Order, Pseudo-Second Order, Intraparticle Diffusio, and Elovich Order, were used to fit the adsorption kinetics process, and three models, e.g., Langmuir, Freundlich, Temkin, and D-R were employed to fit adsorption isotherm.

### Energy of interaction between *B. subtilis* and biochar

To better understand bacterial adhesion on biochar surfaces from the perspective of interfacial interactions and sorption kinetics, the solid surface energy, surface potentials, and interfacial interaction energy between *B.subtilis* and biochar were calculated based on the extended Derjaguin-Landau-Verwey-Overbeek (XDLVO) theory. The detailed calculation procedures are described by Li et al. (2021).

### Salt tolerance test

Pure *B.subtilis* strain and BMFs were inoculated into sterile aqueous solutions at an incubation ratio of 5%, supplemented with 10% salt-free sterilized LB medium. Salt concentrations were set at three gradients: low salt (1%), medium salt (5%), and high salt (10%). The pH were adjusted using dilute using 0.1 mol L^‒1^ HCl or 0.1 mol L^‒1^ NaOH. The cultures were incubated at 30 °C with shaking at 170 rpm for 24 h. Extracellular polymeric substances (EPS) were extracted following the method described by Wang et al. (2023a, b, c), and the contents of proteins, polysaccharides, humic acids, and DNA in EPS were subsequently measured.

### Data analysis

All experimental data was processed using IBM SPSS Statistics (25) (SPSS Inc., Chicago, IL, USA) and Microsoft Excel 2016, and the results were presented as mean ± standard deviation (*n* = 3). Tukey’s T test was employed to evaluate statistical significant, with *p* < 0.05 considered significant. Adsorption experiment data were analyzed and visualized using Origin 2021 (OriginLab, US). Surface characterization data and correlation analyses were also conducted using Origin software. Structural equation model was constructed using the maximum likelihood evaluation program implemented in IBM SPSS AMOS 25 (IBM, USA).

## Results and discussion

### Loading efficiencies of microorganisms on different biochars

**Fig. 1:**
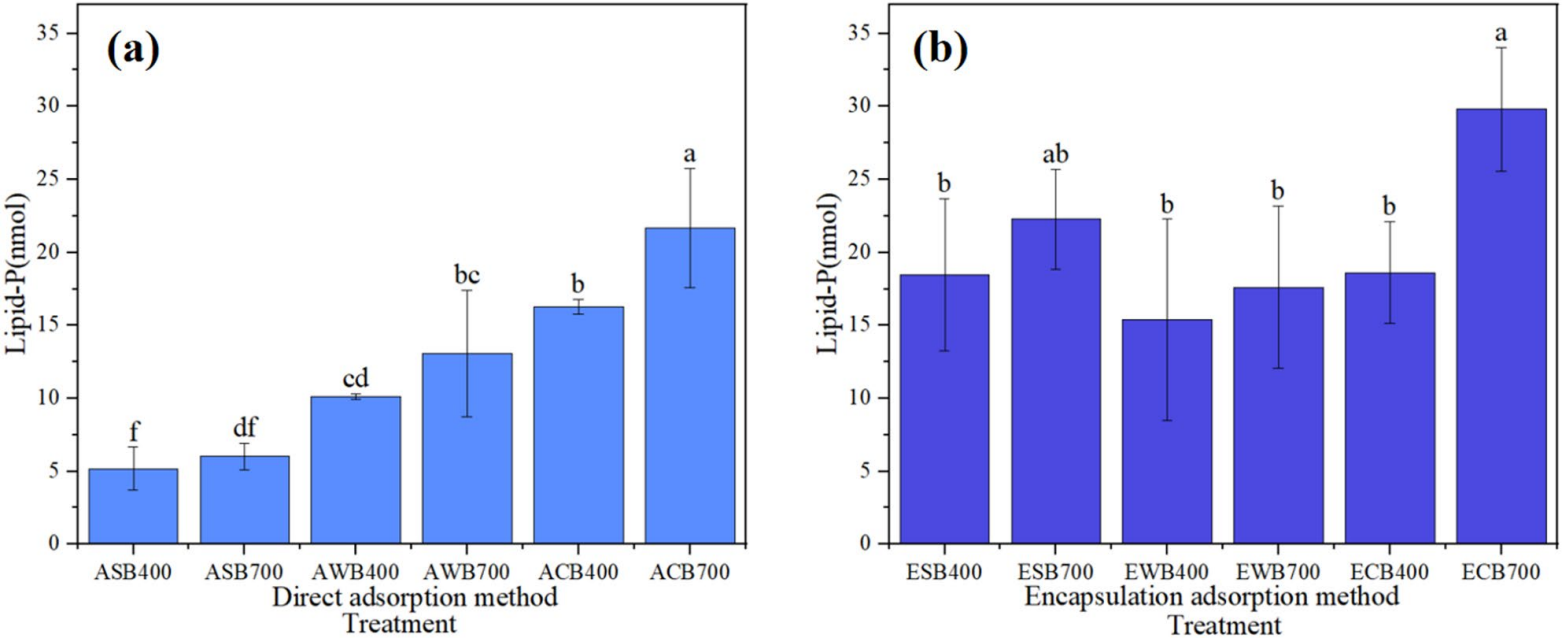
Loading amount of *B.subtilis* on different biochars: (a) adsorption-type BMFs and (b) adsorption-encapsulation-type BMFs. Data are represented as the means ± SD (standard deviation). The different lower case letters indicate significant difference among treatments, which as analyzed by the Tukey’s T test (*p* < 0.05)

The lipid-phosphate method was used to measure the loading of *B.subtilis* on different biochars, and the results are presented in Fig. 1. The loading capacity was found to be significantly affected by the loading method. Using the direct adsorption method, the loading of *B.subtilis* varied markedly among the different biochars (Fig. 1a), whereas only minor differences were observed when the adsorption-encapsulation method was applied (Fig. 1b). Regarding the direct adsorption method, CSB exhibited the highest *B.subtilis* loading (e.g., ACB700 and ACB400), followed by WPB (e.g., AWB700 and AWB400), while SSB (e.g., ASB700 and ASB400) showed the lowest loading capacity (Fig. 1a). For a given feedstock, biochars prepared at high temperature (700 °C) generally had greater *B.subtilis* loading than those produced at lower temperature (400 °C) (Fig. 1a). This difference is likely attributable to the more favorable physical properties of high-temperature biochars, such as larger specific surface area and greater mesopores, and macropore volumes (Table 1) (Yin et al. 2021). For the adsorption-encapsulation method, CSB700 still exhibited the highest *B.subtilis* loading (ECB700), but the differences among biochars were not statistically significant (Fig. 1b). Notably, the loading method had a relatively minor impact on *B.subtilis* loading for CSB and WPB, while the encapsulation approach remarkably improved microbial loading on SSB compared to the direct adsorption method.

### Characterization of biochars before and after *B.subtilis* loading

#### Surface morphology

**Fig. 2:**
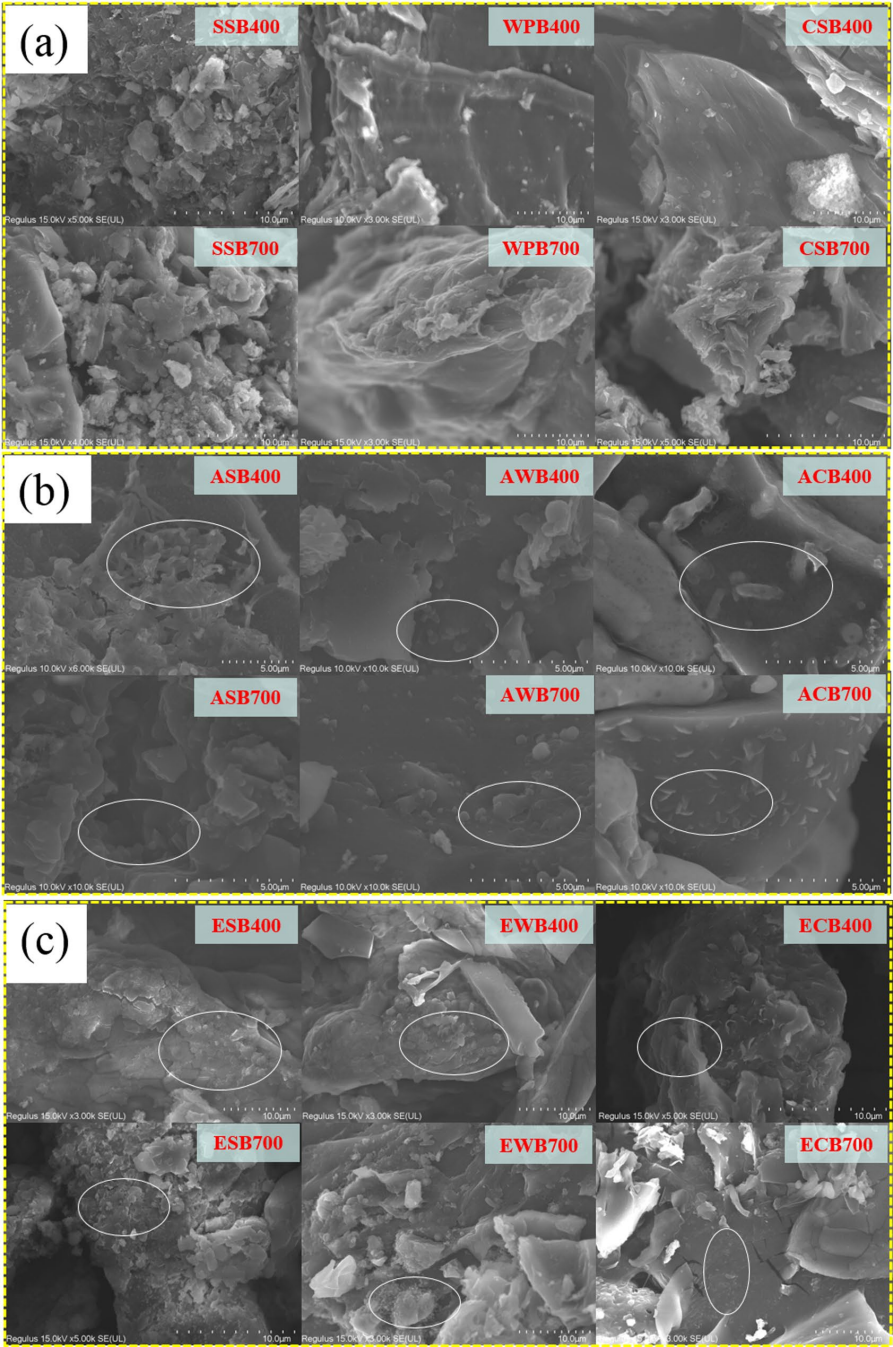
Scanning electron microscopy (SEM) analysis of initial biochars (a), adsorption-type BMFs (b), and adsorption-encapsulation-type BMFs (c)

Significant changes in surface morphology were observed before and after *B.subtilis* loading (Fig. 2). All biochars exhibited large specific surface area and developed porous structure, providing abundant adsorption sites for microorganisms (Liu et al. 2025). As shown in Fig. 2a, the surface of SSB was relatively rough, with numerous protruding particles, likely due to its higher mineral content (Song et al. 2019). For WPB and CSB prepared at low temperatures (400 °C), fibrous structure were observed, which may be attributed to incomplete carbonization of cellulose and lignin in the raw materials (Zhu et al. 2023). At higher temperature (700 °C), pyrolysis promoted the release of volatiles, and restructuring of the carbon matrix, resulting in increased formation of micro- and mesopores and the appearance of surface cracks (Palansooriya et al. 2022). These changes can provide more adsorption sites for microorganisms and additional spaces for metabolite diffusion, supporting microbial growth and metabolic activity (Wang et al. 2025). SEM images confirmed the successful attachment of *B.subtilis* to the biochar surfaces (Figs. 2b and c). Compared to the original biochars, BMFs exhibited clear morphological changes, primarily characterized by the presence of *B.subtilis* cells and their metabolic products distributed on the surface and within pores. *B.subtilis* cells were observed colonizing pores and surface depressions (Fig. 2b), suggesting that initial adhesion likely occurred via pore filling and physical adsorption. *B.subtilis* cells are typically 3–5 μm in length and 1–1.2 μm in width (Liu et al. 2022), and preferentially inhabit pores larger than twice their size (Lehmann et al. 2011; Tao et al. 2018), indicating that mesopores and macropores favor colonization (Tao et al. 2018). Therefore, biochars produced at higher temperatures, which possess improved pore structure, exhibited greater loading capacity (Fig. 1). In addition, EPS secreted by *B.subtilis* partially adhered to the surface of biochar, forming biofilms. Localized aggregation of bacterial cells was also observed, likely due to the present of more functional groups or charged sites that attract cells via electrostatic interaction or hydrogen-binding (Deng et al. 2025). In sum, these observations indicate that microbial loading on adsorption-type BMF is governed by multiple mechanisms. For the adsorption-encapsulation BMFs, it was found that more *B.subtilis* cells were encapsulated in sodium alginate matrix (Fig. 2c). This encapsulation environment limited morphological changes of *B.subtilis* and helped maintain cellular integrity (Sar et al. 2017).

#### Specific surface area and element composition

The specific surface area and elemental analysis of the original biochar are presented in Table 1. Corn straw contains a significant amount of cellulose and hemicellulose, which decompose during pyrolysis, releasing volatile substances and forming a substantial amount of micropores and mesopores (Hu et al. 2021). In contrast, SSBs demonstrated markedly lower specific surface area and pore volume than CSBs and WPBs, which can be attributed to the lower C content of the sewage sludge feedstock and the occurrence of deformation, structure cracking, or micropore blockage (Ippolito et al. 2020; Yin et al. 2021). However, SSB produced at 300 °C was found to possess significantly greater specific surface area and total pore volume than the wood- and reed straw-based biochars produced at the same pyrolysis temperature (Zhu et al. 2026). This unusual phenomenon is related to the high moisture content of the sewage sludge. During low-temperature pyrolysis, the large amount of water vapor generated may interact with organic and inorganic minerals, promoting the release of volatile compounds and the formation of additional pores (Zhu et al. 2026). Both the specific surface area and pore volume of the biochars increased significantly with rising pyrolysis temperature (Table 1). This increase is attributed to the formation of smaller pores resulting from the shrinking of solid matrix at higher temperature (Ippolito et al. 2020). The greater specific surface area and pore volume of the high-temperature biochars suggest that they provide more favorable conditions for microorganism adsorption and colonization.

Biochars produced at high pyrolysis temperature exhibited significantly higher C contents, while greatly lower H, S, and N contents, compared to low-temperature biochars (Table 1). These results are consistent with previous studies, which have shown that increasing pyrolysis temperature promotes the concentration of C element and the volatilization losses of other elements, such as H, O, N, and S (Ippolito et al. 2020; Ortiz et al. 2020; Yin et al. 2021; Zhu et al. 2026). The C/N ratio has been considered a key factor influencing the metabolic activity of microbes (Schommer et al. 2024). CSBs, particularly CSB700, displayed a higher C/N ratio compared to other biochars (Table 1), suggesting that they have more aromatic structures (Uroić Štefanko and Leszczynska 2020). Highly aromatic carbon materials provide adsorption sites through their developed porous structure and surface functional groups, promoting microbial attachment and metabolism (Jia et al. 2020). Furthermore, it has been reported that biochar can regulate the electron transfer process through surface redox-active sites, influencing microbial processes such as nitrification and denitrification (Li et al. 2022).

**Fig. 3:**
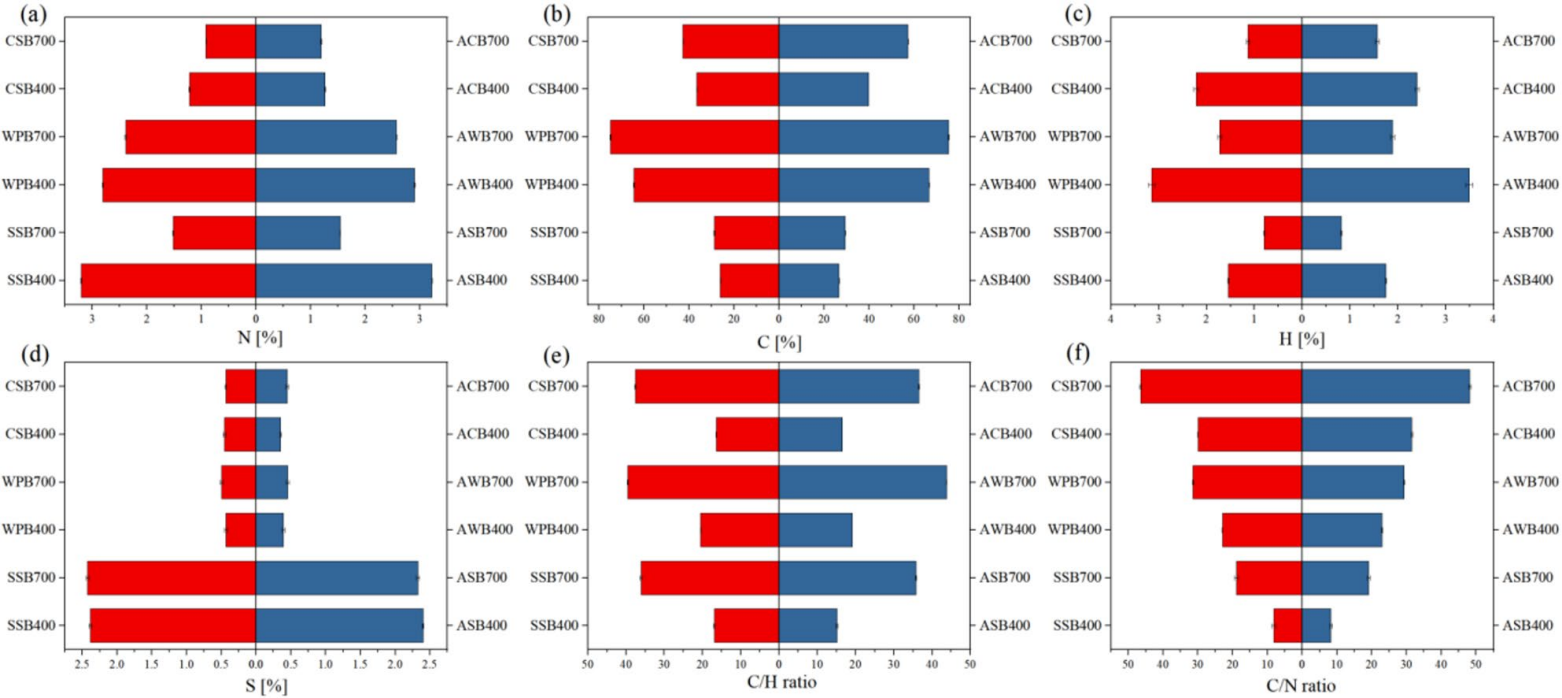
Element composition for original biochars and their corresponding adsorption-type BMFs: (a), N concentration; (b) C concentration; (c) H concentration; (d) S concentration; (e) C/H ratio; and (f) C/N ratio

After *B.subtilis* loading, BMFs demonstrated significant changes in elemental composition (Fig. 3). Compared to the original biochars, C, H, and N contents significantly increased, while S content remarkably decreased for all BMFs (Fig. 3). *B.subtilis* is an N-rich microorganism, with large amounts of N presented in its cell wall and metabolic products (Angeles and Scheffers 2021). This should be the mainly reason for the increased N content. The increases in C and H contents of BMFs may be related to the changes in the surface functional groups and the presence of microbial metabolic products. For example, the loaded *B.subtilis* may produce C- and H-containing metabolic products, such as organic acids, lipopeptides, and polysaccharides. (Schommer et al. 2023).

#### FTIR analysis

**Fig. 4:**
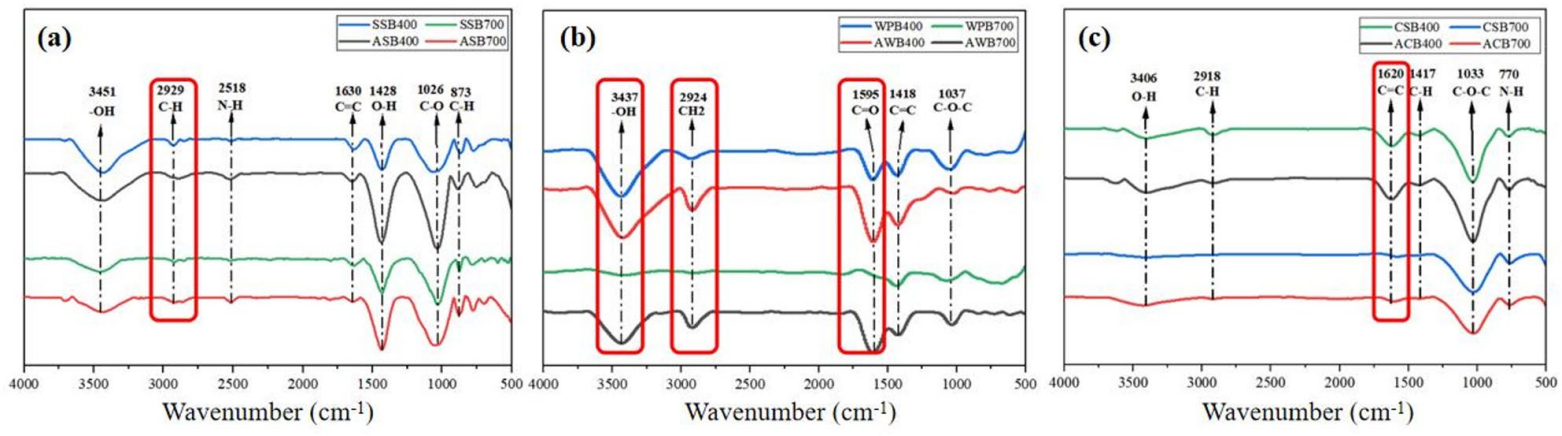
Fourier transform infrared spectroscopy (FTIR) analysis for original biochars and their corresponding BMFs: (a), sewage sludge-derived biochars (SSBs) and BMFs (ASBs); (b) wood power-derived biochars (WPBs) and BMFs (AWBs); and (c), corn stalks-derived biochars (CSBs) and BMFs (ACBs). The red box highlights the functional groups that underwent obvious changes

The differences in the surface functional groups between original biochars and BMFs were analyzed (Fig. 4). Biochars produced from different feedstocks and pyrolysis temperatures exhibited distinct FTIR spectral characteristics (Fig. 4), reflecting differences in the composition of functional groups (Zhang et al. 2024). The surface functional groups of original biochars primarily resulted from the transformation of lignin and cellulose during pyrolysis (Sharma et al. 2023). Increasing pyrolysis temperature reduced the intensity of the O-H stretching vibrations (Fig. 4), likely due to the participation of O-H groups in deoxygenation, decarboxylation, and demethylation reactions at elevated temperatures (Zhang et al. 2024). Regarding CSB and WPB, high-temperature pyrolysis also slightly decreased the intensity of the C-H and C-O-C peaks (Figs. 4b and c), whereas changes in SSB were less pronounced (Fig. 4a). This decrease in O-containing functional groups is consistent with previous studies and is attributed to dehydration and deoxygenation reactions occurring at high pyrolysis temperatures (Lin et al. 2025; Zhu et al. 2026).

After *B.subtilis* loading, all BMFs exhibited increased O-H peak (Fig. 4), which may be due to the interaction between the abundant O-H in the cell wall and membrane of *B.subtilis* and the O-H on the surface of biochar (Ren et al. 2018). The increased intensity of the O-H stretching vibration peak may indicate a potential hydrogen bonding interaction between *B.subtilis* and the biochar surface (Zhang et al. 2024). An enhanced intensity of the C-H stretching vibration peak at 2860–2940 cm^–1^ was also observed for all BMFs (Fig. 4), likely contributed by the aliphatic compounds contained in cell membranes and walls of *B.subtilis* (Ren et al. 2018). Previous study has reported that the alkyl C components contained in *B.subtilis* can interact with the aromatic components of biochar to form adsorption network through hydrophobic interactions (Zhang et al. 2021). Moreover, the peak intensity of C-O group in the 1000–1320 cm^–1^ region increased on the surface of the all of BMFs compared to the original biochars (Fig. 4), which could be originated from polysaccharides within and outside the *B.subtilis* cells (Ren et al. 2018; Xie et al. 2021). Additionally, new C = O peaks observed in AWB (Fig. 4) may be associated with amide groups in microbial proteins (Xie et al. 2021; Ren et al. 2018). A small peak observed around 600–690 cm^–1^ (Fig. 4) may represent typical structural components of EPS (Ren et al. 2018; Xie et al. 2021). Finally, shifts in the signal bands, including 1030 cm^–1^ (C-O stretching), 1440 cm^–1^ (C = C stretching), and 3200 cm^–1^ (O-H stretching), were also found on the surface of BMFs compared to the original biochars (Fig. 4). In summary, the surface functional groups of biochar, especially O-containing groups, changed greatly, indicating that they may play crucial roles in the adsorption of *B.subtilis*. Meanwhile, *B.subtilis* loading on biochar significantly improved the richness and diversity of surface functional groups (e.g., O-H, C-H, C-O, and C = O groups) in the resultant BMFs, which may lead to a greater application potential for soil improvement.

#### Contact angle and Zeta potential

**Fig. 5:**
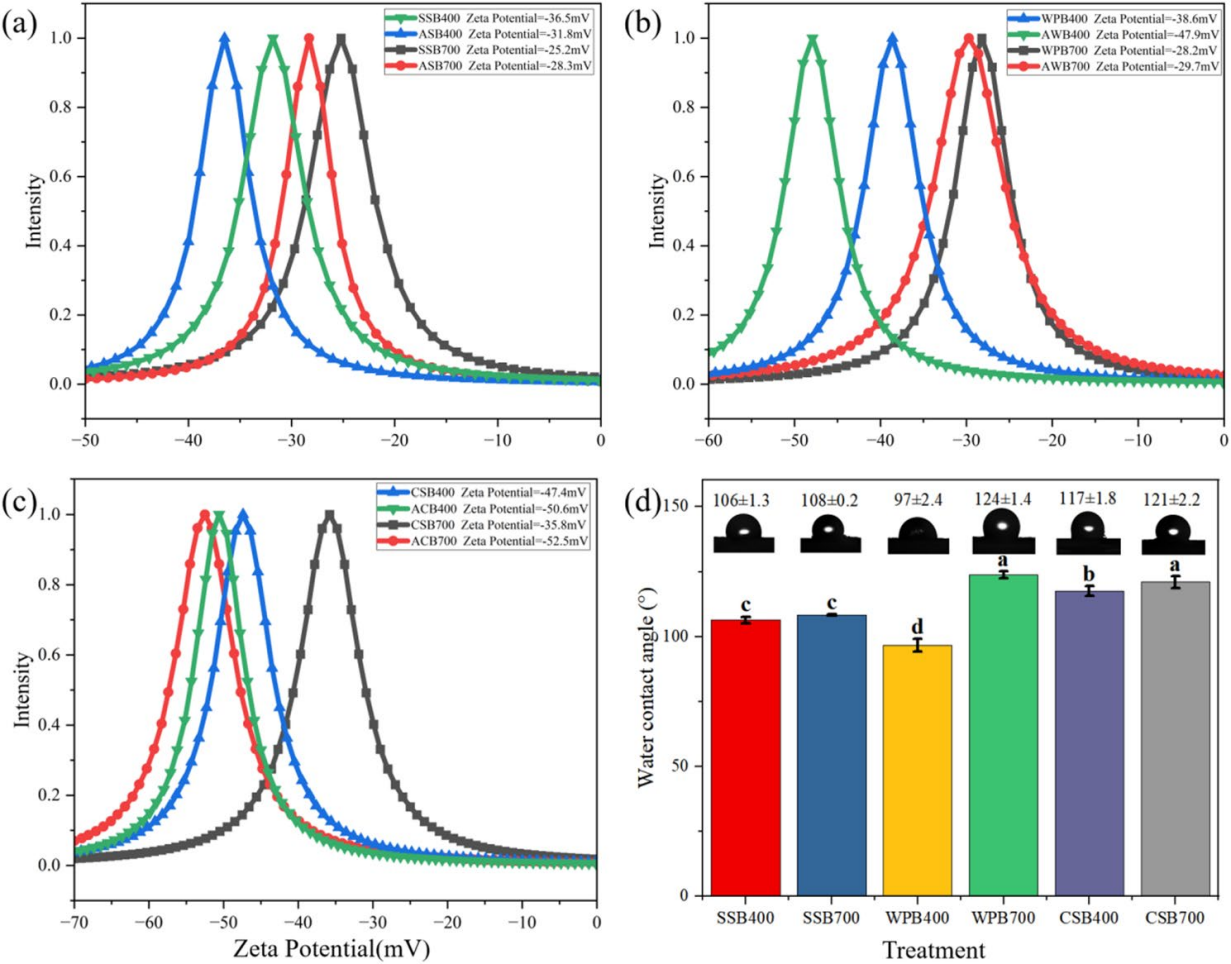
Changes in Zeta potential between original biochars and their resultant BMFs (a–c) and contact angles for the original biochars (d). In panel (d), the different lower case letters indicate significant difference among treatments, which as analyzed by the Tukey’s T test (*p* < 0.05)

Zeta potential is a critical indicator of the surface charge characteristics of particles, with its magnitude directly reflecting the strength of electrostatic interactions between particles (Bejarano et al. 2017). All original biochars exhibited negative Zeta potentials (Figs. 5b–c), imparting a strong negative charge to the biochar surface. *B.subtilis* are also generally negatively charged, because the functional groups such as carboxyl groups on the lipoproteins and lipopolysaccharides on the outer bacterial cell wall carry negative charges (Hrenovic et al. 2009). Therefore, an electrostatic repulsion force should exist between biochar and *B.subtilis*, which may hinder the adsorption process. Compared to the low-temperature biochars, the high-temperature biochars exhibited lower Zeta potentials (Figs. 5b–c), indicating that high-temperature biochars should show a lower electrostatic repulsion with *B.subtilis*. This suggests that high-temperatures biochars are more favorable for *B.subtilis* loading. The Zeta potential of all BMFs remained negative, but the degree of variation varied significantly among different types of biochar (Figs. 5b–c). ACB400 exhibited the greatest difference in the Zeta potential compared to CSB400 (Fig. 5). ASB400 and ACB700 demonstrated the smallest changes compared to their originated biochars (Figs. 5a and c). Although CSB400 exhibited the strongest electrostatic repulsion, supported by the largest absolute value of Zeta potential, it did not have the fewest *B.subtilis* loading amount (Fig. 1). This phenomenon indicates that other non-electrostatic interactions, such as van der Waals forces and Lewis acid-base synergy, played more important roles in the *B.subtilis* adsorption process. These interactions overcame electrostatic repulsion and promoted *B.subtilis* adsorption (Hrenovic et al. 2009).

As illustrated in Fig. 5d, WPB700 and CSB700 were found to be highly hydrophobic, while SSB400 and WPB400 were relatively hydrophilic. The higher the hydrophobicity of the biochar, the more effectively it removes interfacial water, thereby enhancing *B.subtilis* attachment to the biochar surface (Bejarano et al. 2017). Therefore, biochars with higher hydrophobicity, such as CSB700, were consistent with their higher *B.subtilis* loading amounts. For all BMFs, the tested contact angle decreased to 0 (data not shown), indicating an obvious transformation of hydrophobic biochars to hydrophilic BMFs. This change is likely due to the hydrophilic groups presented in *B.subtilis* covering the surface of biochars (Dubois et al. 2020).

### Adsorption behavior of *B.subtilis* on biochars

#### Adsorption kinetics

The adsorption kinetics processes of *B.subtilis* on different biochars were analyzed using the Pseudo-First Order, Pseudo-Second Order, Intraparticle Diffusion, and Elovich Order models, and Elovich models, and the fitting results are presented in Table 2. Among these models, the Pseudo-Second Order model provided the best fit for all biochars (*R*^*2*^ ranged from 0.9986 to 0.9995), suggesting that chemical adsorption was the predominantly process (Wang et al. 2023a, b, c; Mohammed et al. 2018). The Pseudo-First Order model also showed relatively good fitting result (*R*^*2*^ ranged from 0.8851 to 0.9889), indicating that physical adsorption occurred concurrently (Wang et al. 2023a, b, c). As demonstrated by the Elovich Order model, the estimated *α* values were consistently higher than the *β* values across all biochars, implying that the adsorption process occurred more rapidly than desorption (Ton-That et al. 2023).

**Table 2:** Adsorption kinetics of *Bacillus subtilis* on different biochars

Biochar sample	Pseudo-First Order			Pseudo-Second Order			Intraparticle Diffusio						Elovich		
	Qe	K1	*R* ^2^	Qe	K1	*R* ^2^	Kid1	Kid2	I1	I2	R1^2^	R2^2^	α	β	*R* ^2^
SSB400	69.7	0.158	0.99953	70.7	0.012	0.9996	15.25	0.126	1.69	68.60	0.9578	0.7269	347,705	4.42	0.98604
SSB700	77.4	0.127	0.99497	79.2	0.007	0.99998	15.66	0.128	1.85	76.52	0.9521	0.9220	5766	6.87	0.98101
WPB400	71.1	0.171	0.99883	72.0	0.014	0.99985	15.65	0.057	2.13	70.85	0.9374	0.9845	2,756,357	3.97	0.98977
WPB700	76.1	0.152	0.99088	78.4	0.007	0.99981	15.90	0.307	2.56	73.85	0.9132	0.3024	44,512	5.68	0.99166
CSB400	71.4	0.152	0.99984	71.8	0.014	0.99928	15.75	0.023	1.29	71.00	0.9766	0.3781	348,395	4.52	0.97763
CSB700	80.0	0.148	0.99825	81.4	0.009	0.99992	17.07	0.132	2.00	79.02	0.9531	0.4134	77,253	5.71	0.98559

*Note: Qe*, *K1*, and *R*^*2*^ in represent the equilibrium absorption capacity, rate constant, and fitting coefficient, respectively; *Kid1* and *Kid2*, *I1* and *I2*, and *R1*^*2*^ and *R2*^*2*^ denote the two-stage intraparticle diffusion rate constants, constants related to the boundary layer thickness, and fitting coefficients, respectively; *α*, *β*, and *R*^*2*^ in Elovich model indicate initial adsorption rate, a constant associated with surface heterogeneity and the activation energy of chemisorption, and fitting coefficient, respectively. Abbreviations: SSB400 and SSB700, sewage sludge biochars produced at 400 °C and 700 °C, respectively; WPB400 and WPB700, wood power biochars produced at 400 °C and 700 °C, respectively; CSB400 and CSB700, corn stalks biochars produced at 400 °C and 700 °C, respectively

#### Adsorption isotherm

The adsorption isotherms of *B.subtilis* on varied biochar were fitted by the Langmuir, Freundlich, Temkin, and D-R models, with the results presented in Table 3. The Langmuir model manifested the best fitting result (*R*^*2*^ ranged from 0.9432 to 0.9978), supporting that the adsorption process may be monolayer adsorption on a uniform surface (Mohammed et al. 2018). It should be noted that Freundlich parameter *n* ranged from 1.3361 to 1.9746, indicating favorable adsorption and suggesting that physical interactions, such as Van der Waals’ force, pore filling, and electrostatic attraction), contribute to the process (Wang et al. 2023a, b, c). However, the relatively high fitting coefficients of Freundlich model (*R*^*2*^ = 0.9435–0.9908) also imply that physical adsorption is not the only mechanism. The Temkin isotherm model, which describes adsorption behavior on heterogeneous surfaces (Tounsadi et al. 2016), also showed good agreement with the experimental data for most biochars (*R*^*2*^ = 0.9403–0.9984), except for WPB700. These results further highlight the complex and multi-mechanistic nature of *B.subtilis* on biochar (Tounsadi et al. 2016).

**Table 3:** Adsorption isotherms of *Bacillus subtilis* on different biochars

Biochar sample	Langmuir			Freundlich			Temkin			D−*R*		
	Q_max_ (CFU g^−1^)	b	*R* ^2^	K_F_	*n*	*R* ^2^	AT (L g^−1^)	βT (kJ mol^−1^)	*R* ^2^	q_mD−*R*_ (mg g^−1^)	E (kJ mol^−1^)	*R* ^2^
SSB400	73.06	0.0026	0.9904	34.66	1.3706	0.9808	0.3807	2.24	0.9714	74.75	7502.49	0.8562
SSB700	84.56	0.0053	0.9802	53.51	1.5059	0.9604	0.5163	1.93	0.9753	82.01	2723.98	0.8835
WPB400	79.28	0.0052	0.9925	45.37	1.6112	0.9908	0.5012	1.66	0.9403	78.60	4491.91	0.7923
WPB700	86.01	0.0055	0.9432	56.98	1.5456	0.9435	0.6030	1.55	0.8161	91.12	3705.60	0.7564
CSB400	79.00	0.0026	0.9907	37.37	1.3361	0.9811	0.4101	2.25	0.9880	74.47	5501.99	0.8909
CSB700	80.41	0.0142	0.9978	63.60	1.9746	0.9836	0.6675	1.47	0.9984	72.53	537.80	0.8159

*Note: Q*_*max*_ and *b* represent the maximum adsorption capacity and the Langmuir affinity constant, respectively; *K*_*F*_ and *n* are the Freundlich constants reflecting adsorption capacity and intensity, respectively; *AT* and *βT* are Temkin constants, with *βT* related to the heat of adsorption; *q*_*mD−R*_ and *E* denote the maximum adsorption capacity and mean free energy in the D-R model, respectively; and *R*^*2*^ represents the fitting coefficient. Abbreviations: SSB400 and SSB700, sewage sludge biochars produced at 400 °C and 700 °C, respectively; WPB400 and WPB700, wood power biochars produced at 400 °C and 700 °C, respectively; CSB400 and CSB700, corn stalks biochars produced at 400 °C and 700 °C, respectively

### Interface interaction

The surface wettability of the six original biochars was evaluated by contact angle measurements, using formamide, diiodomethane, and water as probe liquids. The results demonstrated that, except for CSB, all biochars exhibited a contact angles of 0° with diiodomethane, indicating complete wettability. In contrast, CSB displayed non-zero contact angles for all three liquids, consistent with its superior *B.subtilis* loading performance (Fig. 1). Therefore, subsequent analyses only focused on the interfacial interaction mechanisms of CSB.

**Fig. 6:**
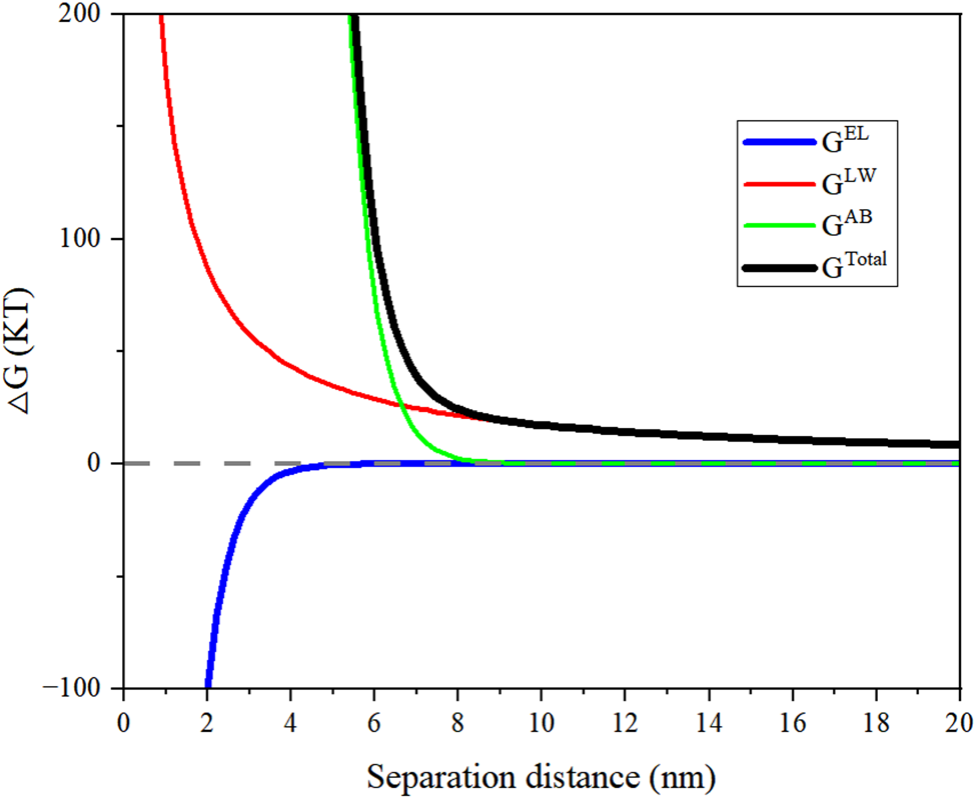
Binding energy (ΔG) for *B.subtilis* adsorbed to biochar surface analyzed by extended Derjaguin-Landau-Verwey-Overbeek (XDLVO) theory. The curves demonstrate the van der Waals energy (ΔG^LW^), electrical double-layer energy (ΔG^EL^), acid-base energy (ΔG^AB^), and total interaction energy (ΔG^TOT^) as a function of the separation distance measured by its surface parameter and Zeta potential

According to the extended XDLVO theory, the total interfacial free energy (ΔG_*TOT*_) is the sum of the Lewis acid-base free energy (ΔG_*AB*_), van der Waals free energy (ΔG_*LW*_), and electrostatic double-layer free energy (ΔG_*EL*_) (Li et al. 2021). As illustrated in Fig. 6, the variation of these interaction energies with separation distance aligned with XDLVO theory predictions. The van der Waals force (ΔG_*LW*_) monotonically decreased with increasing distance, meaning that it is a primary attractive force at short range (Brosh et al. 2014). The electrostatic force (ΔG_*EL*_) exhibited repulsion at short distances but decayed rapidly with increasing separation (Li et al. 2021).The Lewis acid-base interaction energy (ΔG_*AB*_), often associated with hydrophobic interactions, showed strong attraction at short distances, likely due to reduced exposure of hydrophobic regions on the biochar surface (Dubois et al. 2020). The total interaction energy (ΔG_*TOT*_) indicated that spontaneous attraction occurs between biochar and *B.subtilis* at short separation distances. This attraction is primarily driven by the combined effects of hydrophobic interactions (ΔG_*AB*_), van der Waals force (ΔG_*LW*_), and electrostatic attraction (ΔG_*EL*_) (Li et al. 2021).

### Correlation analysis and adsorption mechanisms

**Fig. 7:**
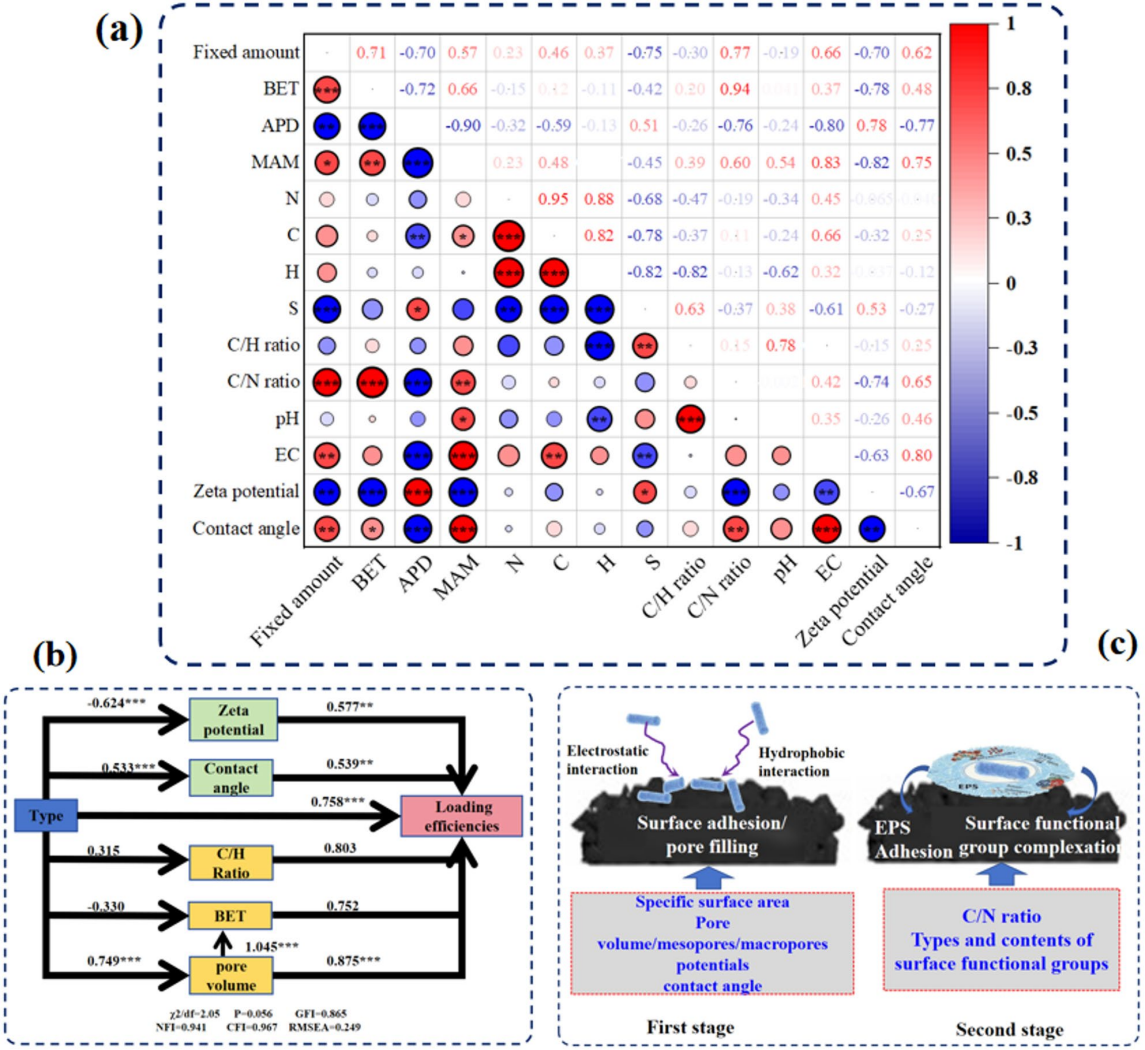
Correlation analysis between *B.subtilis* loading amount and physicochemical properties of original biochar (a), acting pathways of biochar type and key properties on *B.subtilis* loading amount (b), and two-stage adsorption process of *B.subtilis* on biochar and the key biochar properties (c). Abbreviations: BET, specific surface area; APD, average pore diameter; MAM, volume of mesopores and macropores; EC, electrical conductivity. *, **, and *** in panel (a) indicate *p* < = 0.05, <= 0.01, and < = 0.001, respectively

To further explore the key biochar properties governing *B.subtilis* adsorption, correlation analyzes were performed between the physicochemical properties of biochar and the *B.subtilis* loading capacity. As illustrated in Fig. 7a, B.*subtilis* loading was significantly positively correlated specific surface area, average pore diameter, and volumes of mesopore and macropore. This result confirms the crucial role of biochar physical structure in *B.subtilis* adsorption. In contrast, *B.subtilis* loading exhibited a significant negative correlation with Zeta potential of biochar, likely due to the stronger electrostatic repulsion at higher surface charge. Furthermore, significant positive correlations were identified between *B.subtilis* loading and the C/H and C/N ratios of biochar, meaning that chemical composition also plays an important role in the adsorption process (Yu et al. 2024; Schommer et al. 2024). Biochars with higher C/N or C/H ratios typically exhibit higher EC, which may provide more ion-exchange sites for *B.subtilis* attachment (Zhang et al. 2022). Moreover, enhanced conductivity can facilitate interspecies electron transfer, which is crucial for microbial metabolic activity and biofilm formation (Chen et al. 2014). Therefore, *B.subtilis* loading was also significantly positive correlated with EC. A significant negative correlation was observed between *B.subtilis* loading and S content, which may be ascribed to the inhibitory or acidifying effects of sulfur-containing compound on microbial activity (Wang et al. 2023a, b, c). Finally, *B.subtilis* loading was positively correlated with contact angle. The surface with a higher hydrophobicity may trigger a higher Lewis acid-base interaction, synergistically drives the emptying of water molecules at the interface (Dubois et al. 2020), which will be conducive to the contact between microbe and biochar. Structural equation model further confirmed that *B.subtilis* loading was directly influenced by Zeta potential, contact angle, C/H ratio, specific surface area, and pore volume, with standardized path coefficients of 0.577, 0.539, 0.803, 0.752, and 0.875, respectively (Fig. 7b).

In summary, the adsorption of *B.subtilis* on biochar is a dynamic process involving multiple coordinated mechanisms, which can be conceptualized as two consecutive stages (Fig. 7c). In the initial stage, physical interactions predominately drive the adsorption process, as supported by the results derived from Pseudo-First order model. During this stage, *B.subtilis* rapidly adheres to the biochar surface through electrostatic interactions and pore filling. Therefore, biochar properties such as specific surface area, pore volume―particularly mesopores/macropores―Zeta potential, and hydrophobicity can significantly affect the efficiency of *B.subtilis* adsorption. In the subsequent stage, chemical adsorption gradually becomes the dominant, as indicated by the results derived from Pseudo-Second order model, with stable bonding forming between *B.subtilis* and the surface functional groups of biochar. EPS produced by *B.subtilis* may further contribute to the stable adhesion of bacteria. In this stage, biochar characteristics such as C/N ratio and the abundance of surface functional groups are the key factors determining the stable immobilization of *B.subtilis*. Based on these mechanistic insights, an ideal biochar carrier for *B.subtilis* should possess the following characteristics: high specific surface area and porosity, near-neutral surface charge, high C/N ratio, and good conductivity. Therefore, biochars derived from cellulose-rich feedstock and high pyrolysis temperature (e.g., CSB700) are particularly recommended.

### Salt tolerance test

**Fig. 8:**
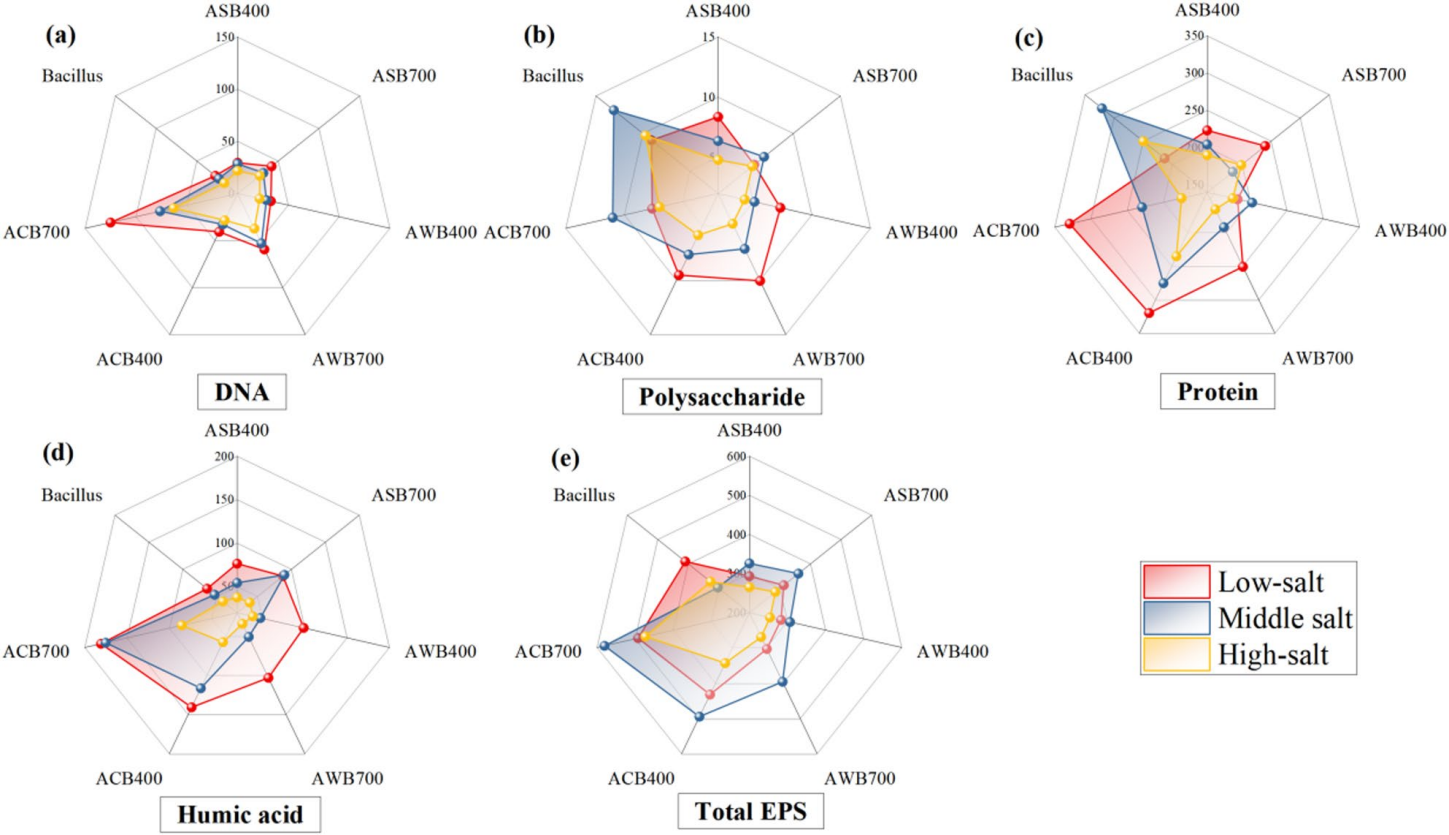
Production amounts of DNA (a), polysaccharide (b), protein (c), humic acid (d), and total extracellular polymeric substance (EPS) (e) under different salinity levels for different BMFs

The salt tolerance of *B.subtilis* before and after immobilization on different biochars was measured to evaluate the potentials of biochar carriers in improving microbial salt tolerance. As demonstrated in Fig. 8, the salt tolerance of the biochar-immobilized *B.subtilis* varied significantly across different BMFs. As salinity increased, DNA content decreased in all BMFs, likely due to the enhanced inhibitory effect of salt ions on *B.subtilis* activity, which impacts microbial colonization and survival (Boyandin et al. 2000). Compared to pure *B.subtilis*, the salt tolerance of *B.subtilis* immobilized on biochar was significantly improved, although the improvement degree varied across different biochars. The DNA contents of ACB700 observed under different salinity conditions were consistently significantly higher than other BMFs (Fig. 8a), indicating a higher *B.subtilis* activity and proliferation. A similar variation was observed for humic acid content, which decreased with increasing salinity across all BMFs (Fig. 8d). Humic acid, as a natural chelating agent, can be combined with cationic salt to reduce the direct toxic effect of salt on cells (Krachler et al. 2019). Therefore, the high humic acid content in ACB700 (Fig. 8d) suggests an enhanced resistance to salt stress in immobilized *B.subtilis*. Under high or extreme salinity conditions, microorganisms may secrete more EPS to provide a physical barrier, reducing the potential for salt ion entry into cells (Cam and Badilli 2023). Microorganisms may also secrete more proteins under different salinity conditions to maintain the stability of the extracellular environment (Cam and Badilli 2023). Hence, the increased protein content under high or extreme salinity environments may reflect the bacterium’s adaptation to high osmotic pressure by secreting protective proteins to prevent dehydration and damage (Feng et al. 2022). Under extreme salinity conditions, all BMF, except for AWB400, displayed an increase in protein content, particularly ACB700 and ACB400, supporting that biochar as a carrier promoted *B.subtilis* to secrete more proteins to withstand extreme salt stress. Notably, pure *B.subtilis* had a significantly higher protein content than BMFs under low and high salinity conditions (Fig. 8c). This may be due to a disturbance by the protein and polysaccharide contained in the nutrient solution used during the experiment. Due to the higher activity of *B.subtilis* in the BMF treatments, it is reasonable inferred that the protein and polysaccharide in the nutrient solution could be consumed more rapidly. However, polysaccharide content did not increase in the BMF treatments compared to the pure *B.subtilis* treatment, and even showed a decreasing trend in certain BMF treatments (Fig. 8b). This outcome indicates that biochar may have little influence on *B.subtilis* to secrete additional polysaccharides to counteract salt stress. EPS secretion is a critical defense strategy for microorganisms under salt stress (Khan et al. 2019). Specifically, tightly bound EPS (TB-EPS) can protect microorganisms from high salt concentrations (Feng et al. 2022). It has been reported that the EPS produced by *Alcaligenes Latus* can absorb up to 1000 times its dry weight in water, thus maintaining stable intracellular conditions under high salt concentrations (Boukhelata et al. 2018; Vu et al. 2009). EPS has also been shown to enhance microbial salt tolerance by regulating proline production or uptake and Na^+^ accumulation (Mukherjee et al. 2019). Compared to other BMFs and pure *B.subtilis*, ACB700 and ACB400 exhibited significantly higher total EPS contents (Fig. 8e). This result supports the conclusion that the salt tolerance of *B.subtilis* can be more efficiently improved using CSB as a carrier, particularly when produced at high-temperature (CSB700). In conclusion, CSB700 not only exhibited the highest *B.subtilis* loading capacity, but also demonstrated the greatest potential for enhancing *B.subtilis* salt tolerance. Therefore, ACB700 is highly recommended as the desired BMF in saline-alkali soil improvement. However, it should be emphasized that determining the optimal BMF type solely based on salinity tolerance tests is inadequate, and it is necessary to establish a more robust evaluation framework from a practical application perspective—for instance, through soil incubation or pot experiments in saline-alkaline soils, incorporating different treatments (untreated, *B. subtilis* alone, biochar alone, *B. subtilis* + biochar, and BMF) to systematically compare the effects of various amendments on soil properties and plant growth.

## Conclusions

This study systematically investigated the immobilization performance and underlying mechanisms of *B.subtilis* on various biochars. Our findings demonstrated that both feedstock type and pyrolysis temperature significantly affected *B.subtilis* immobilization performance, primarily through their decisive effects on biochar properties (e.g., physical structure, surface chemistry). Based on assessments of loading capacity and salt tolerance, biochars derived from corn straw and produced at high temperatures (e.g., CSB700) were recommended as optimal carriers for *B.subtilis* attachment. Further analysis revealed that the immobilization of *B.subtilis* on biochar was co-contributed by multiple mechanisms, including physical adsorption controlled by pore filling, electrostatic interaction, and complexation driven by surface functional groups. Therefore, more precise control over biochar surface characteristics―such as increasing the abundance of mesopores and macropores and enhancing surface positive charge―could further improve the immobilization efficiency of *B.subtilis*. Nonetheless, additional direct evidence, such as that from complementary surface-sensitive analyses, is required to more clearly elucidate the immobilization mechanisms and quantitatively distinguish the contributions of each. Overall, this study provides novel insights into the immobilization mechanisms of *B.subtilis* on biochars and offers strong support for the development of efficient biochar-based microbial fertilizers (BMFs).

## Data Availability

All data are available within the article and are also available from the corresponding author upon reasonable request.
